# Quantifying the human milk oligosaccharides 2’‐fucosyllactose and 3‐fucosyllactose in different food applications by high‐performance liquid chromatography with refractive index detection

**DOI:** 10.1111/1750-3841.15005

**Published:** 2020-01-22

**Authors:** Anne Støvlbæk Christensen, Sabina Holm Skov, Sara Eun Lendal, Bettina Høj Hornshøj

**Affiliations:** ^1^ DuPont Nutrition Biosciences ApS Edwin Rahrs Vej 38 8220 Brabrand Denmark

**Keywords:** 2’‐fucosyllactose, 3‐fucosyllactose, high‐performance liquid chromatography, milk, yoghurt

## Abstract

**Abstract:**

In recent years, production of biosynthesized human milk oligosaccharides (HMOs) has become scalable to industrial standards. As a result, infant formula fortified with 2’‐fucosyllactose (2’‐FL), the most abundant HMO in human breast milk, is now commercially available. 2’‐FL and 3‐fucosyllactose (3‐FL), another abundant HMO, are thought to be beneficial for infant health and development. Products containing HMOs are projected to expand in the future, showing the need for robust, easily applicable analytical methods for the quantitative assessment of HMOs in different food applications. We present here a validated high‐performance liquid chromatography method for the quantification of 2’‐FL and 3‐FL in whole milk, infant formula, and cereal bars. The sample preparation was simple dispersion and extraction of the sample. The samples were analyzed by hydrophilic interaction liquid chromatography with refractive index detection and a runtime of 19 min. The method had a high degree of linearity (*R* ^2^ > 0.9995) in the range 0.2 to 12 mg/mL. The recovery for 2’‐FL was 88% to 105% and for 3‐FL 94% to 112%. The limit of detection (LOD) for whole milk was 0.1 mg/mL for 2’‐FL and 0.2 mg/mL for 3‐FL. In infant formula and cereal bars, the LOD was 0.6 mg/g for both 2’‐FL and 3‐FL. To show the practical application of this method, it was successfully utilized in stability studies of 2’‐FL and 3‐FL in whole milk, UHT milk, and yoghurt. The method provides a means of simultaneous and robust quantification of 2’‐FL and 3‐FL in various food matrices with high accuracy and high reproducibility.

**Practical Application:**

2’‐Fucosyllactose (2’‐FL) and 3‐fucosyllactose (3‐FL) are two of the most abundant human milk oligosaccharides (HMOs) present in human breast milk. We present a fast HPLC method for the robust quantification of these two compounds in infant formula, whole milk, UHT milk, cereal bars, and yoghurt. This method can easily be set up by food producers and researchers to analyze the dosage of 2’‐FL and 3‐FL in their product or perform shelf life studies in different food applications.

## 1 INTRODUCTION

Human milk oligosaccharides (HMOs) are a diverse class of oligosaccharides and glycoconjugates present in human breast milk. Chemically, HMOs are built from five monosaccharides: galactose, glucose, N‐acetylglucosamine, fucose, and N‐acetyl‐neuraminic acid. More than 150 HMO compounds have been structurally described to date (Urashima, Hirabayashi, Sato, & Kobata, 2018).

The nutritional benefits of HMOs include supporting the development of the infant gut microbiota as bifidogenic prebiotics (Garrido, Dallas, & Mills, 2013; Thomson, Medina, & Garrido, 2018), attenuating food‐related allergies (Zehra et al., 2018), and promoting brain development (Oliveros et al., 2016; Wang, 2012). Only approximately 1% of HMOs are absorbed into systemic circulation and later eliminated with the urine (Goehring, Kennedy, Prieto, & Buck, 2014; Rudloff, Pohlentz, Borsch, Lentze, & Kunz, 2011). The bulk of HMOs are either metabolized by bacteria or excreted in the feces (Davis et al., 2016).

**Figure 1:**
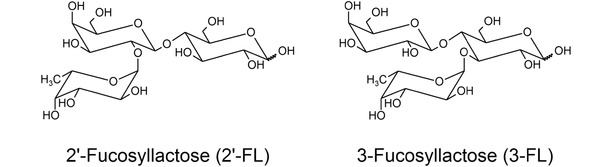
Structure of 2’‐fucosyllactose (2’‐FL) and 3‐fucosyllactose (3‐FL).

The concentration of HMOs ranges from approximately 20 mg/mL in colostrum to 5 to 15 mg/mL in mature milk (Elwakiel et al., 2018; Gabrielli et al., 2011; N. Sprenger, Lee, De Castro, Steenhout, & Thakkar, 2017; Xu et al., 2017). 2’‐Fucosyllactose (2’‐FL; Figure 1) is the most abundant HMO in both colostrum and mature milk, with concentrations of 0.76 to 4.78 mg/mL (Thurl et al., 2010; Thurl, Munzert, Boehm, Matthews, & Stahl, 2017). 3‐Fucosyllactose (3‐FL; Figure 1) is one of the most abundant HMOs after 2’‐FL, with concentrations of 0.3 to 0.58 mg/mL in breast milk (Austin & Benet, 2018; Balogh, Szarka, & Beni, 2015; Thurl et al., 2017).

It has been shown that infant formula containing 2’‐FL and lacto‐*N‐*neotetraose (LNnT) supported age‐appropriate growth, lower rates of both morbidity and medication use (Puccio et al., 2017). Adults supplemented with 2’‐FL and LNnT displayed increased levels of *Bifidobacterium* and no adverse effects (Elison et al., 2016), showing a potential for HMOs in nutrition beyond infant formula.

In recent years production of biosynthesized HMOs has become scalable to industrial standards (G. A. Sprenger, Baumgartner, & Albermann, 2017) and infant formula fortified with 2’‐FL is now commercially available. With the portfolio of products containing HMOs projected to expand in the future, there is a need for robust, easily applicable methods for qualitative and quantitative assessment of biosynthesized HMOs.

The most frequently used methods for quantification of HMOs are based on liquid chromatography (LC). As oligosaccharides do not contain fluorophores or chromophores, chemical modification of HMOs is often employed to improve detection and chromatographic retention, especially with fluorescence and ultraviolet detectors (Ruhaak & Lebrilla, 2012). For increased sensitivity and structural information, LC can be coupled to a mass spectrometer (MS; Yan, Ding, & Liang, 2017). Multiple reaction monitoring (MRM) has increasingly been used as the technique that allows for sensitive (fmol range) and fast quantitation of HMOs (Hong et al., 2014; Mank, Welsch, Heck, & Stahl, 2019).

Reversed‐phase high‐performance liquid chromatography (RP‐HPLC) and porous graphitized carbon (PGC) as column stationary phase are routinely used in combination with MS (Balogh, Jankovics, & Beni, 2015; Bao, Chen, & Newburg, 2013; Dong, Zhou, & Mechref, 2016; Oursel, Cholet, Junot, & Fenaille, 2017; Tonon, Miranda, Abrao, de Morais, & Morais, 2019). PGC chromatography provides good resolution and can separate oligosaccharide isomers, even the α and β anomeric configuration (Bao et al., 2013). However, elution of anomers complicates the interpretation of the chromatogram. PGC chromatography typically requires a reduction and salt removal step of samples to obtain separation of HMOs, but sensitivity is high (Grabarics, Csernak, Balogh, & Beni, 2017). LC‐MS systems are often expensive to acquire and maintain, and therefore not standard equipment in analytical laboratories.

Capillary electrophoresis (CE) and gas chromatography (GC) are techniques with high sensitivity and resolution used for quantification of HMOs. However, these techniques often require derivatization, complicating the sample preparation (Balogh et al., 2015; Galeotti et al., 2014; Mantovani, Galeotti, Maccari, & Volpi, 2018).

High‐performance anion‐exchange chromatography coupled with pulsed electrochemical detection is a well‐established and sensitive method for HMO analysis (Kunz, Rudloff, Hintelmann, Pohlentz, & Egge, 1996) with good separation of structural isomers such as 2’‐FL and 3‐FL (Yan et al., 2017). However, dedicated equipment is required due to the highly basic mobile phase used.

Hydrophilic interaction liquid chromatography (HILIC) is an established method for analysis of both underivatized and derivatized carbohydrates in food matrices (Marrubini, Appelblad, Maietta, & Papetti, 2018). HILIC can be combined with a broad range of detectors, such as MS, UV (ultraviolet), evaporative light scattering (ELS), and refractive index detection (RI), although RI is only suitable for isocratic methods (Buszewski & Noga, 2012; Wuhrer, de Boer, & Deelder, 2009).

All the methods mentioned above for the analysis of HMOs, often require extensive sample preparation procedures, long runtimes, combination of instrument platforms, sometimes very expensive equipment, and are aimed at elucidating structural and biological functions.

The goal of this study was to implement a simple, fast, and easy applicable method on relatively inexpensive equipment for the quantification of 2’‐FL and 3‐FL in different food applications. The HPLC instrument with an RI detector was selected as it is relatively inexpensive and often present in food producer's quality control laboratories, at universities, and research sites.

The method presented here is based on separation on a HILIC column for the absolute quantification of 2’‐FL and 3‐FL. To represent different food applications, the method was developed for analysis of milk‐based products, cereal‐based products, and dietary supplements. For the validation study, whole milk was selected as representative for UHT milk and yoghurt, infant formula as a representative for powder based dietary supplements, and cereal bars representing cereal‐based products.

As an example of practical application, the method was utilized in stability studies of 2’‐FL and 3‐FL in whole milk, UHT milk, and yoghurt. To the best of our knowledge, this is the first validated method for absolute quantification of 2’‐FL and 3‐FL in different food applications using HPLC‐RI.

## 2 MATERIALS AND METHODS

### 2.1 Chemicals and standards

HPLC grade acetonitrile and isopropanol were purchased from Thermo Fisher Scientific. Triethylamine (≥99%) was obtained from Sigma‐Aldrich. 2’‐FL (>95%) was purchased from Carbosynth (Berkshire, UK). The standard for 3‐FL (99.7%) was kindly provided by Danisco Sweeteners Oy (Kantvik, Finland). Lactose monohydrate and sucrose were both purchased from Merck (Darmstadt, Germany). Water was purified in a Milli‐Q water purification system from Millipore (Molsheim, France).

### 2.2 Samples

Whole milk samples with and without 2’‐FL and 3‐FL were produced at DuPont (Brabrand, Denmark) using standard recipes. Infant formula and cereal bar samples with and without 3‐FL were produced at DuPont (New Century, Kansas, USA) using standard recipes. Infant formula with 2’‐FL was a Similac^®^Pro‐Advance HMO infant formula (Abbott). For the stability study whole milk, UHT milk, and yoghurt samples with and without 2’‐FL and 3‐FL were produced at DuPont (Brabrand, Denmark), using standard recipes.

### 2.3 Standard solution preparation

A stock solution was prepared by weighing out and dissolving 2’‐FL and 3‐FL in water (12 mg/mL). Standard solutions were made by serial dilution in the concentration range 0.2 to 6.6 mg/mL in water.

A 16 mg/mL solution of sucrose and lactose was used as retention time reference standard only. For recovery studies, a spiking solution containing 5 mg/mL of 2’‐FL or 3‐FL was used.

### 2.4 Sample preparation

**Figure 2:**
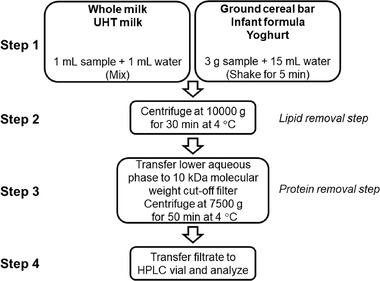
Sample preparation workflow.

All samples were prepared in triplicate unless otherwise noted. If the whole milk was not received fresh (i.e., frozen), it was defrosted at room temperature and the milk placed at 5 °C for 24 hours to let the pH stabilize from pH 6 to neutral (pH 7). The sample preparation workflow is shown schematically in Figure 2 and described in further detail here. One milliliter of water was added to 1 mL milk (whole milk or UHT milk) and the mixture centrifuged at 10,000 *g* for 30 min at 4 °C (Thermo Fisher Scientific Heraus Multifuge X3R, Osterode, Germany). The lower aqueous phase was transferred to a 10 kDa molecular weight cut‐off filter (Vivacon 500, Sartorius, Stonehouse, UK) and centrifuged for 50 min at 7,500 *g* at 4 °C. The filtrate was transferred to an HPLC vial and was ready for analysis.

Cereal samples were ground by a blender (Philips, HR1375, Brazil). Three grams of either ground cereal bar, infant formula, or yoghurt samples were added to a plastic tube and 15 mL of water was added. The samples were shaken for 5 min (Heidolph Multi Reax Shaker, Schwabach, Germany) and then centrifuged at 10,000 *g* for 30 min at 4 °C. The lower aqueous phase was treated as above.

For recovery studies 2’‐FL and 3‐FL solutions were added at step 1 in the sample preparation workflow (Figure 2).

### 2.5 HPLC‐RI analysis

The HPLC‐RI analysis of 2’‐FL and 3‐FL was performed on an Agilent/Hewlett Packard HPLC system equipped with a degasser (G1379B, Japan), binary pump (G1312B), micro well plate autosampler (G1377A), thermostat for autosampler (G1330A), column compartment (G1316B), and a refractive index detector (G1362A; all units except G1379B from Germany). The compounds were separated on an Acquity UPLC^®^ BEH Amide column (Waters, Dublin, Ireland, 50 × 2.1 mm i.d., 1.7 µm) equipped with a Vanguard Acquity UPLC^®^ BEH Amide guard column (Waters, 5 × 2.1 mm i.d., 1.7 µm) using isocratic elution with a mobile phase consisting of acetonitrile, water, and triethylamine (785/215/5, v/v/v). The flow was 0.2 mL/min and the runtime 19 min. The autosampler was kept at 10 °C and the injection volume was 2 µL. The needle was washed for 3 s in the flush port with a mixture of water and isopropanol (900/100, v/v) for each injection. The column oven was set at 40 °C as was the refractive index detector.

### 2.6 Method validation

The validation of this HPLC‐RI method was performed according to the principles in the ICH Harmonised Tripartite Guideline (ICH, 2005). The steps that were evaluated for the validation were specificity, linearity, accuracy, precision (repeatability and intermediate precision), robustness, and detection/quantification limit. The samples used for the validation were whole milk with and without 2’‐FL and 3‐FL, infant formula, and cereal bars with and without 3‐FL, and infant formula with 2’‐FL.

## 3 RESULTS AND DISCUSSION

### 3.1 Robustness

To obtain separation between 2’‐FL, 3‐FL, and an unknown peak which was present in the infant formula and cereal bar samples, the following mobile phase compositions were tested: acetonitrile/water/triethylamine 742/275/10, 750/250/10, 775/225/10, 780/220/10, 785/215/10, and 790/210/10 (v/v/v). The mobile phases containing 742, 750, 775, and 780 mL acetonitrile were not suitable for separating 2’‐FL, 3‐FL, and the unknown peak, but 785 and 790 mL acetonitrile provided an improved resolution. It was chosen to use 785 mL acetonitrile to allow more water in the mobile phase. Decreasing the triethylamine to 5 mL resulted in a higher signal intensity, as did increasing the injection volume from 1 to 2 µL (data not shown). Hence, the final mobile phase composition providing the best separation was acetonitrile/water/triethylamine (785/215/5, v/v/v) with an injection volume of 2 µL.

The robustness of the method was initially tested by changing the temperature of the column while keeping the temperature of the RI detector constant at 40 °C. A high concentration standard containing sucrose, lactose, 2’‐FL, and 3‐FL (concentration: 17, 15, 11, and 12 mg/mL, respectively) was analyzed at five different column temperatures (25, 35, 40, 45, and 50 °C) and the resolution between the peaks were calculated (Table 1).

**Table 1:** Resolution between peaks in standard of sucrose, lactose, 2’‐FL and 3‐FL at five different temperatures

	Resolution (*R*_s_)	
Temperature (°C)	Lactose‐2’‐FL	2’‐FL‐3‐FL
25	2.35	0.81
35	2.06	0.82
40	1.78	0.85
45	1.96	0.81
50	2.09	0.87

The resolution between lactose and 2’‐FL was above 1.5 for all temperatures tested. The best resolution between 2’‐FL and 3‐FL was obtained at 50 °C, namely 0.87. However, to extend the lifetime of the guard column and column, the lower temperature with the second‐best resolution, 40 °C, was chosen for the final method. Overall, the resolution between the peaks did not change significantly, showing that the method is robust towards change in the temperature of the column. The 2’‐FL and 3‐FL peaks were not baseline separated, but as described in the specificity section the resolution was found to be acceptable for quantification of 2’‐FL and 3‐FL in the different food application samples.

Regarding the day‐to‐day runs, it is important to monitor the pressure of the system, as this is one of the primary indicators for changing the guard column. Although the sample preparation workflow has a step for both lipid and protein removal from the matrix of the samples (Figure 2), the simplicity of the procedure still allows some components to adhere to the column material. This results in shifts in retention time between sequences, an increase in pressure over time or an increase in the level of noise. However, the resolution between 2’‐FL, 3‐FL, and the unknown does not change significantly with a deteriorating column. Depending on the runs, we have seen that the guard column needs to be changed after approximately 200 injections. Because of the increase in pressure over time, the flow rate of the method was kept at 0.2 mL/min. A lower flow rate would increase runtime, which was not desirable. The flow rate could be increased if an ultra‐high‐performance liquid chromatography system was used, but the intention was to employ a relatively inexpensive HPLC equipment to allow a broader use of the method.

### 3.2 Specificity

**Figure 3:**
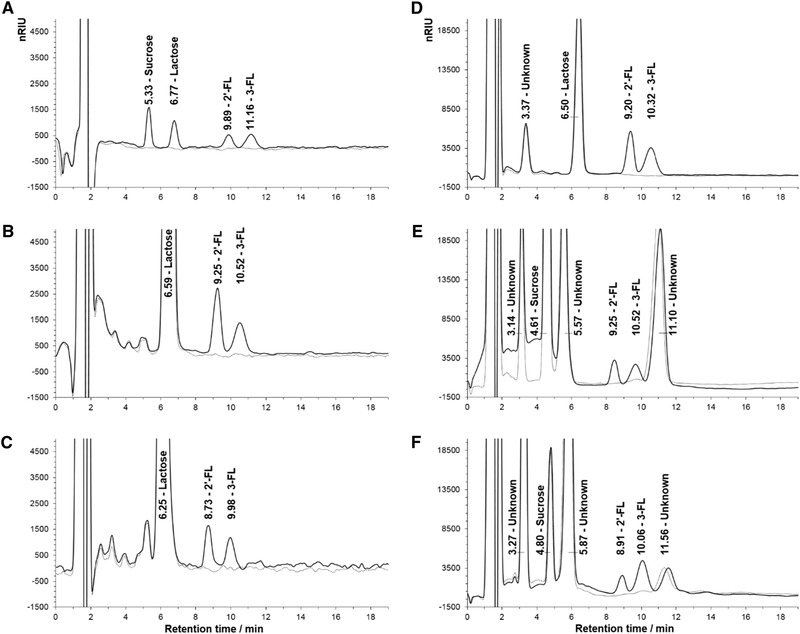
Magnified view of chromatograms of lowest standard of 2’‐FL and 3‐FL including sucrose and lactose (A), whole milk containing 2’‐FL spiked with 3‐FL (B), UHT milk containing 2’‐FL and 3‐FL (C), yoghurt containing 2’‐FL and 3‐FL (D), infant formula containing 3‐FL spiked with 2’‐FL (E), and cereal bars containing 3‐FL spiked with 2’‐FL (F). Dotted lines are blank chromatograms for the corresponding matrices (i.e. water (A), whole milk (B), UHT milk (C), yoghurt (D), infant formula (E), and cereal bars (F)). nRIU, nano refractive index units.

The specificity was determined by the resolution between 2’‐FL and 3‐FL and the surrounding peaks in the chromatogram (Table 2). In Figure 3, the chromatogram of a standard of sucrose, lactose, 2’‐FL, and 3‐FL are shown together with the chromatograms of the respective food matrices with 2’‐FL and 3‐FL, all overlaid with the corresponding blank matrix (dotted lines). The resolution between 2’‐FL and earlier eluting compounds (lactose or unknown) was 1.75 to 2.95, between 2’‐FL and 3‐FL 0.95 to 1.05, and between 3‐FL and later eluting unknown it was 0.95 to 1.00 depending on the matrices. Hence, there was a good baseline separation between 2’‐FL and earlier eluting compounds, but between 2’‐FL, 3‐FL, and the unknown later eluting peak there was not a baseline separation (usually indicated by a resolution >1.5). 2’‐FL and 3‐FL are added in relatively low dosages in the different food matrices (in this case approx. 1 mg/mL in whole milk and 6 to 9 mg/g in infant formula and cereal bars). Hence, the risk of coelution affecting quantification is minimal. If 2’‐FL and 3‐FL were added in larger amounts, this could affect the quantification. As described in the accuracy section, the recoveries of 2’‐FL and 3‐FL were acceptable, indicating that coelution was not a problem in this analysis. If available, a blank matrix can be run to check for coelution. With these precautions, the resolution was found to be satisfactory for the quantification of 2’‐FL and 3‐FL.

**Table 2:** Resolution between peaks in whole milk, infant formula, and cereal bar

	Resolution (*R*_s_)			
Sample	Lactose‐2’‐FL	Unknown‐2’‐FL	2’‐FL‐3‐FL	3‐FL‐unknown
Whole milk	1.75	–	0.95	–
Infant formula	–	2.68	1.05	0.95
Cereal bar	–	2.95	0.97	1.00

Note: “**–**“ indicates no peak (lactose or unknown) present in matrix.

### 3.3 Linearity, limit of detection (LOD) and limit of quantification (LOQ)

The linearity of the method was tested in the range of 0.2 to 12 mg/mL for 2’‐FL and 3‐FL. Standard solutions were prepared on three different days and run in 4 to 11 replicates. The data for the calibration curves for the three different days is shown in Table 3. The residual sum of squares for the calibration curves for each day were all >0.9995 indicating a high degree of linearity in the tested range.

**Table 3:** Calibration curve and residual sum of squares from linear regression for 2’‐FL and 3‐FL

Compound	Calibration curve^a^	RSQ^b^
2’‐FL	(101.3 ± 3.0)× – (7215 ± 1891)	0.9997 ± 0.0002
3‐FL	(103.8 ± 3.0)× – (10008 ± 2344)	0.9997 ± 0.0002

a Five data points (*n* = 3), *y* = (*α* ± SD)*x* – (*β* ± SD), where SD is the standard deviation; *x* is the nominal concentration in mg/mL of 2’‐FL and 3‐FL, respectively; and *y* is the area of 2’‐FL and 3‐FL, respectively.

b RSQ, residual sum of squares.

The limit of detection and limit of quantification were determined from a signal‐to‐noise ratio of 3 and 10, respectively, by spiking 2’‐FL and 3‐FL in low concentrations in whole milk, infant formula, and cereal bars which did not already contain 2’‐FL and 3‐FL. The signal‐to‐noise ratio was determined by relating the height of the signal for 2’‐FL and 3‐FL to the height of the noise surrounding 2’‐FL and 3‐FL in the chromatograms.

For whole milk, the limit of detection was 0.1 mg/mL for 2’‐FL and 0.2 mg/mL for 3‐FL. The limit of quantification was 0.4 and 0.7 mg/mL for 2’‐FL and 3‐FL, respectively. In infant formula and cereal bars, the limit of detection was 0.6 mg/g and the limit of quantification was 2 mg/g for both 2’‐FL and 3‐FL.

### 3.4 Accuracy

The accuracy was assessed using 12 determinations over 4 concentration levels for both 2’‐FL and 3‐FL (Table 4). The spike levels for milk were approx. 0%, 50%, 100%, and 150% of an anticipated dosage level of 1 mg/mL. For infant formula and cereal bars, the spike levels were approx. 0%, 25%, 50%, and 80% of an anticipated dosage level of 6 mg/g. Whole milk, infant formula, and cereal bar samples were spiked with a solution containing 5 mg/mL of either 2’‐FL or 3‐FL. All samples were spiked at step 1 in Figure 2.

**Table 4:** Recovery of 2’‐FL and 3‐FL in whole milk, infant formula, and cereal bar spiked with solutions of 2’‐FL and 3‐FL

	2’‐FL		
Sample	Target spike level^a^	Recovery curve, *R* ^2^	Recovery (%)
Whole milk with 3‐FL	Low	1.0483*x* + 0.033, *R* ^2^ = 0.9944	105
Whole milk with 2’‐FL	Low	1.0062*x* + 1.1088, *R* ^2^ = 0.9979	101
Infant formula with 3‐FL	High	0.8831*x* + 0.0309, *R* ^2^ = 0.9972	88.3
Infant formula with 2’‐FL	High	0.8876*x* + 6.5619, *R* ^2^ = 0.9979	88.8
Cereal bar with 3‐FL	High	0.9141*x* + 0.0579, *R* ^2^ = 0.9961	91.4

	3‐FL		
Sample	Target spike level^a^	Recovery curve, *R* ^2^	Recovery (%)
Whole milk with 3‐FL	Low	0.9405*x* + 1.1063, *R* ^2^ = 0.9873	94.1
Whole milk with 2’‐FL	Low	1.1159*x* + 0.064, *R* ^2^ = 0.9884	112
Infant formula with 3‐FL	High	0.9662*x* + 6.4599, *R* ^2^ = 0.9717	96.6
Infant formula with 2’‐FL	High	0.9432*x* + 0.0721, *R* ^2^ = 0.9962	94.3
Cereal bar with 3‐FL	High	0.9582*x* + 8.9265, *R* ^2^ = 0.9723	95.8

a Target spike level: Low = 0, 0.5, 0.9, 1.4 mg/mL; High = 0, 1.5, 3.0, 4.5 mg/g.

The recovery for 2’‐FL was 88% to 105% and for 3‐FL 94% to 112% (Table 4). The AOAC guideline for dietary supplements states that an acceptable recovery at 1 mg/mL level is 90% to 108% (AOAC, 2013). Hence, the method presented here has an acceptable recovery.

### 3.5 Repeatability and intermediate precision

The repeatability of the method was investigated using six determinations of each sample type performed by one analyst (*n* = 6). The intermediate precision of the method was investigated using six determinations of each sample type performed on two different days by two different analysts (*n* = 12).

The average concentration for each sample type along with the standard deviation, relative standard deviation (RSD%), and the 95% confidence interval for both repeatability and intermediate precision are shown in Table 5.

**Table 5:** Repeatability and intermediate precision for whole milk, infant formula, and cereal bar

	Whole milk		Infant formula		Cereal bar
Repeatability (*n* = 6)	2’‐FL (mg/mL)	3‐FL (mg/mL)	2’‐FL (mg/g)	3‐FL (mg/g)	3‐FL (mg/g)
Average	1.13	1.17	6.61	6.21	8.81
SD^a^	0.05	0.06	0.13	0.25	0.41
RSD^b^ (%)	4	5	2	4	5
95% CI^c^	0.04	0.05	0.10	0.20	0.33
**Intermediate precision (*n* = 12)**					
Average	1.11	1.13	6.65	6.24	8.66
SD^a^	0.04	0.08	0.12	0.25	0.36
RSD^b^ (%)	4	7	2	4	4
95% CI^c^	0.02	0.05	0.07	0.14	0.20

a SD, standard deviation.

b RSD, relative standard deviation.

c 95% CI, 95% confidence interval.

The relative standard deviation for repeatability and intermediate precision for whole milk, infant formula, and cereal bar samples containing 2’‐FL was <5%. For infant formula and cereal bar samples containing 3‐FL, the relative standard deviation for repeatability and intermediate precision was also <5%. However, for whole milk sample containing 3‐FL, the relative standard deviation for repeatability and intermediate precision was slightly higher at 5% to 7%. Overall the method showed a satisfactory precision.

### 3.6 Stability study for 2’‐FL and 3‐FL in whole milk, yoghurt, and UHT milk

A stability study for 2’‐FL and 3‐FL in pasteurized whole milk and yoghurt at 5 °C was set up for 22 and 36 days, respectively. These durations were chosen as the typical shelf‐life for whole milk and yoghurt is 2 to 4 weeks and 3 to 4 weeks, respectively. The milk was produced containing 1 mg/mL and the yoghurt containing 10 mg/g of both 2’‐FL and 3‐FL. Similarly, a stability study of 2’‐FL and 3‐FL in UHT milk at room temperature for 3 months was also conducted. It was chosen to conduct the study for the first half of the shelf‐life for UHT milk, which is typically >6 months. The UHT milk was produced containing 1 mg/mL of 2’‐FL and 3‐FL.

An initial recovery study was performed in yoghurt where 2’‐FL and 3‐FL were spiked in yoghurt not containing 2’‐FL and 3‐FL. The recovery for 2’‐FL and for 3‐FL was 83%, which was acceptable.

**Figure 4:**
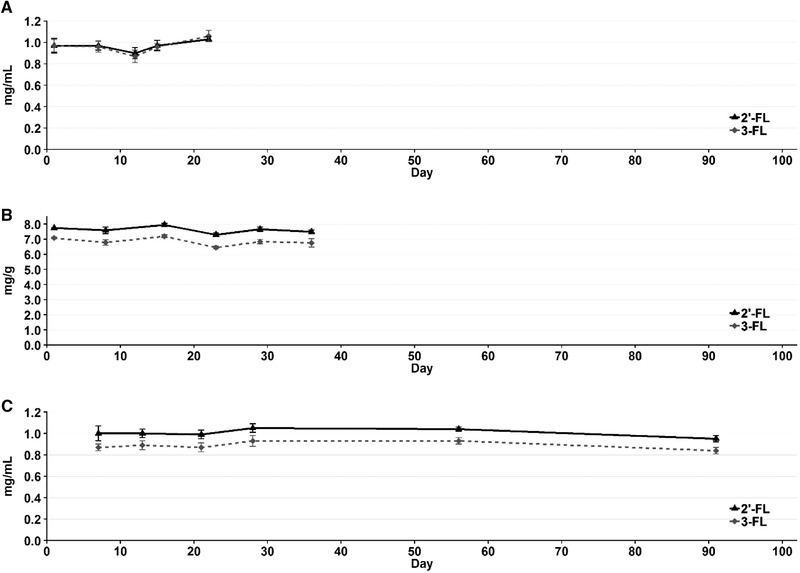
Stability study of 2’‐FL and 3‐FL in whole milk (A), yoghurt (B), and UHT milk (C) shown with the corresponding error bars (standard deviation, *n* = 3) for each measurement.

The stability study data can be seen in Figure 4 and shows that over the course of the stability study for both whole milk, yoghurt, and UHT milk, 2’‐FL and 3‐FL are stable. However, the start dosage in yoghurt was 10 mg/g, but only a mean (for all measurements in the study) of 8 mg/g 2’‐FL and 7 mg/g of 3‐FL was recovered in the yoghurt. Our literature study did not yield any comprehensive data on the thermal stability of 2’‐FL and 3‐FL at elevated temperatures, but we hypothesize that the higher and longer pasteurization temperature used to produce the yoghurt or the fermentation process and the lower pH of the yoghurt could be the reason for this decrease. A lower content of 3‐FL in UHT milk was also observed, which could indicate that 3‐FL is not as stable as 2’‐FL during the production of the samples.

## 4 CONCLUSION

A validated, highly robust, and reproducible HPLC‐RI method for the quantification of 2’‐FL and 3‐FL has been established according to the ICH guideline principles. As an HPLC instrument with refractive index detection is a relative inexpensive equipment, it is often present at food producer's quality control laboratories, universities, and research facilities and therefore, the method presented in this paper can easily be set up and used to check the dosage of 2’‐FL and 3‐FL in different food applications or perform shelf life studies.

## AUTHOR CONTRIBUTIONS

Anne Støvlbæk Christensen performed most of the analytical work for this paper. Sabina Holm Skov and Anne Støvlbæk Christensen designed the HPLC‐RI method and sample preparation procedures. Sara Eun Lendal did the literature study and wrote the introduction. Bettina Høj Hornshøj wrote the remainder of the manuscript, prepared tables and graphics and did the analytical work for intermediate precision as analyst number two.

## CONFLICTS OF INTEREST

The authors declare that they have no conflict of interest regarding the publication of this paper.
